# Comparative efficacy of three active treatment modules on psychosocial variables in patients with long-term mechanical low-back pain: a randomized-controlled trial

**DOI:** 10.1186/s40945-015-0010-0

**Published:** 2015-09-21

**Authors:** Chidozie Emmanuel Mbada, Olusola Ayanniyi, Samuel Olusegun Ogunlade

**Affiliations:** 1 Department of Medical Rehabilitation, College of Health Sciences, Obafemi Awolowo University, Ile-Ife, Nigeria; 2 African Population and Health Research Center, Nairobi, Kenya; 3 Department of Physiotherapy, College of Medicine, University of Ibadan, Ibadan, Nigeria; 4 Department of Orthopaedic and Trauma, College of Medicine, University of Ibadan, Ibadan, Nigeria

**Keywords:** McKenzie protocol, Fear-avoidance behaviour, Pain self-efficacy belief, Back pain belief, Muscles endurance exercise

## Abstract

**Background:**

Psychosocial factors precipitate and perpetuate the risk of developing long-term Low-Back Pain (LBP) with resultant disability. However, management of psychosocial aspects of LBP still remains a major challenge. This study investigated the effect of static or dynamic back extensors endurance exercise on psychosocial variables of Fear-Avoidance Behaviour (FAB), Pain Self-Efficacy Belief (PSEB) and Back Pain Consequences Belief (BPCB) in patients with LBP.

**Methods:**

A randomized-controlled trial of 67 patients assigned into McKenzie Protocol (MP) group (*n* = 25), MP and Static Endurance Exercise Group (MPSEEG; *n* = 22); and MP and Dynamic Endurance Exercise Group (MPDEEG; *n* = 20) was carried out. Treatment was applied thrice weekly for eight weeks.

**Results:**

The groups were comparable in general and baseline psychosocial parameters (*p* > 0.05). The different regimens had significant effects on all outcome parameters across baseline, 4^th^ and 8^th^ week (*p* < 0.05). The regimens were comparable in mean change scores on BPCB and FAB at the 4th and 8th week respectively (*p* > 0.05). MPDBEEG had higher mean change in PSEB at the 4^th^ and 8^th^ week respectively.

**Conclusions:**

McKenzie Protocol alone, or in combination with static or dynamic back extensors endurance exercise has comparable effect on FAB, PSEB and BPCB in patients with LBP. The addition of dynamic endurance exercise to the MP led to significantly higher positive effects on PSEB.

**Electronic supplementary material:**

The online version of this article (doi:10.1186/s40945-015-0010-0) contains supplementary material, which is available to authorized users.

## Background

Low-Back Pain (LBP) is a constellation of symptoms of pain or discomfort resulting from mulfactorial aetiology [1, 2] with anatomical, physiological, psychological and social consequences [2, 3]. LBP as a complex disorder occurs in a wide variety of medical, musculoskeletal, and neurologic conditions [3] and it is often classified as acute, sub-acute and chronic according to duration of pain [4]. Chronic LBP is defined as spinal pain persisting for at least twelve weeks [5]. It is believed that the word “chronic” may be associated with negative expectations, therefore, based on the International Classification for Functioning, Disability and Health (ICF) framework, the word “long-term” is preferred [6].

Long-term LBP results in both physical and psychological deconditioning that traps the patient in a vicious circle characterized by decreased physical performance, exacerbated nociceptive sensations, depression, impaired social functioning, and work disability [7]. The multifactorial biopsychosocial problem associated with long-term LBP include fear of movement, anxiety, and faulty coping strategy, low self-efficacy, poor self-esteem and disability [8–11]. However, whether these psychosocial factors are causes or consequences of LBP has been the subject of debate [12]. Management of LBP is described as a continuum of physical and psychosocial factors, with varying amounts of each [13]. The traditional approach based on a biomedical model is centered on the treatment of physical factors [13]. Now, it is widely accepted that LBP and disability can only be understood and managed in the light of a biopsychosocial model (a model that includes physical, psychological and social elements), which describes the key psychological and behavioural factors that may help to understand current levels of pain and disability [13, 14].

Systematic reviews of the evidence concerning the effectiveness of exercise concluded that exercise may be helpful for patients with long-term LBP in terms of decrease in pain and disability [15], decrease in psychosocial dysfunctions such as fear of avoidance behaviour [16, 17]. Consequently, exercises of various types have been used in managing LBP with varying reported successes [18] as they appear to be the central element in the physical therapy management of patients with long-term mechanical LBP [15, 19, 20]. Still, there seems to be no consensus on the most effective exercise programme for patients with LBP. One of the most commonly used methods of evaluation and treatment among physiotherapists is the McKenzie method [21]. However, the effectiveness of the McKenzie method on bio-behavioral factors such psychosocial and cognitive variables in patients with LBP is still contentious [22, 23]. On the other hand, there is emerging evidence to suggest that endurance training of the low-back extensors can be effective in addressing the multifactorial physiological and biopsychosocial problem in patients with LBP [24–26]. Therefore, this study aimed to test the hypothesis that there would be no significant difference in the effects of static or dynamic back extensors endurance exercise in combination with McKenzie Protocol on psychosocial variables of fear-avoidance behaviour, pain self-efficacy belief and back pain consequences belief in patients with long-term mechanical LBP.

## Methods

### Participants

**Fig. 1:**
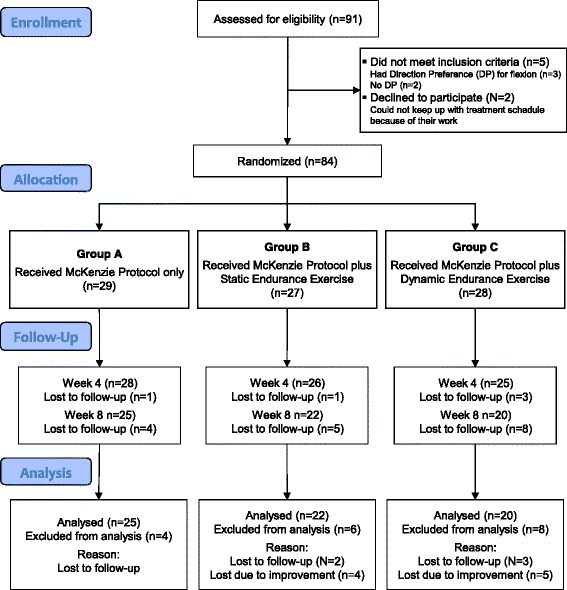
Flow of participants through the study

A total of 84 consecutive patients recruited from the Out-patient Physiotherapy Department of the Obafemi Awolowo University Teaching Hospitals Complex (OAUTHC); and the Department of Medical Rehabilitation, Obafemi Awolowo University, Ile-Ife, Nigeria met the inclusion criteria for this study. The patients were randomly assigned to one of three treatment groups; the McKenzie Protocol (MP) Group (MPG) (*n* = 29), MP plus Static Back Endurance Exercise Group (MPSBEEG) (*n* = 27) and MP plus Dynamic Back Endurance Exercise Group (MPDBEEG) (*n* = 28). However, 67 patients (25, in MPG, 22 in MPSBEEG and 20 in MPDBEEG) completed the eight weeks study with a drop-out rate of 20.2 %. Based on Cohen [27], using 0.05 α level, degree of freedom of 2, effect size of 0.25, and power of 80, a minimum sample size of 52 was adopted for this study. The CONSORT showing the recruitment, assignment and progression of patients through the study is presented in Fig. 1.

Blinding was introduced in order to reduce bias. A research assistant co-ordinated the recruitment, eligibility screening and assignment of the patients into the different treatment groups (A, B, or C). In order to ensure equal-sized treatment groups, random permuted blocks was used and a block size of 6 was chosen (i.e. AABBCC, ABABCC and all the other possible restricted permutations). The block permutations were computer-generated using a factorial equation formula: (6!) / ((2!)(2!)(2!)) = 90. The process of drawing block permuted sequence and randomization was repeated as the participants were recruited. The research assistant was not involved in the treatment of the participants. The researchers (CEM and OA) are credentialed in the McKenzie Diagnosis and Therapy and solely supervised the protocols.

Eligibility to participate in this study was determined using the McKenzie Institute’s Lumbar Spine Assessment Format (MILSAF) [28]. Long-term mechanical LBP was defined as back pain of not less than three months which was of musculoskeletal origin and in which symptoms vary with physical activity [29]. Based on the MILSAF, patients who demonstrated “Directional Preference” (DP) for extension only were recruited in order to ensure homogeneity of samples. DP is described as the posture or movement that reduces or centralizes radiating pain that emanates from the spine. Apart from excluding patients with DP for flexion, lateral or no DP, other exclusion criteria were a positive history of red flags indicative of serious spinal pathology (with complaints of dermatomal sensory loss, myotomal muscle weakness and reduced lower limb reflexes), any obvious spinal deformity or neurological disease; pregnancy; previous spinal surgery; and previous experience of static and dynamic endurance exercise. Participants in this study were exclusively on physical therapy and were admonished not to seek other treatment or self therapy for their LBP without informing the researchers.

Anthropometrical parameters of the participants, such as height and weight were measured following standard procedures. Body mass index was calculated as the ratio of weight in kilograms to height squared. Ethical approval for the study was obtained from the joint UI/UCH Ethical Review Committee (Ref no.: UI/UC/10/0194) and the Ethics and Research Committee of the Obafemi Awolowo University Teaching Hospitals Complex (Ref no.: ERC/2010/01/02).

### Instruments

*Pain Self-Efficacy Questionnaire* (PSEQ) was used to assess the self-efficacy beliefs specifically related to basic physical activities. This 7-point Likert scale (where 0 = not at all confident and 6 = completely confident) of ten items covers a range of functions, including household chores, socializing, work as well as coping with pain without medications was developed by Nicholas [30]. Participants were asked to rate how confidently they can perform the activities described, at present, despite their pain. A summative total score, ranging from 0 to 60, was calculated. Higher score on the scale reflects a stronger self-efficacy belief [30, 31]. High scores (>40) indicate the client is likely to respond well to an exercise program. Low scores (<20) indicate the client is more focused on the pain. A Yoruba version of the PSEQ was used for participants who were literate in the Yoruba language and preferred the Yoruba version. Pearson product moment correlation coefficient (r) of 0.82 was obtained for the criterion validity of the back translation of the Yoruba version.

*Fear-Avoidance Beliefs Questionnaire* (FABQ) was used to assess pain-related fear of physical activity that causes avoidance of activity and increased disability. FABQ developed by Waddell et al. [32], measures pain-related fear of physical activity that causes avoidance of activity and increased disability. It has a minimum score of 0 and a maximum score of 42 from the 7-items scale. The higher the scale scores the greater the degree of fear and avoidance beliefs shown by the patient. A Yoruba version of the FABQ was also used in this study. Pearson product moment correlation coefficient (r) of 0.80 was obtained for the criterion validity of the back translation of the Yoruba version.

*The Back Beliefs Questionnaire (BBQ)* was used to assess general beliefs about the inevitable consequences of future life with LBP [12]. This tool consists of 14 statements to which the participants indicated their level of agreement on a 5 point scale on beliefs about pain and its consequences. A score of 1 indicates complete disagreement and a score of 5 complete agreement. As 5 of the 14 statements are distractors, the scores of the 9 remaining statements are reversed and then summed to provide a total score ranging from 9 to 45. A lower score indicates the respondent has more negative beliefs about back pain. A Pearson product moment correlation coefficient (r) of 0.79 was obtained for the criterion validity of the back translation of the Yoruba version used in the study.

*The Oswestry Low-Back Disability Questionnaire* (OLBPQ) was used to assess disability (i.e. “the limitations of a patient’s performance”) [33]. OLBPQ covers 10 domains including pain intensity, personal care, lifting, walking, sitting, standing, sleeping, sex life, social life and traveling [33]. For each domain there is a scale of six statements (score 0–5), where zero is the ability to perform the activity without pain and five is inability to perform the activity because of pain. Therefore, higher score means high degree of activity limitation. The disability sum score is calculated as total score divided by total possible score and multiply by 100 [33]. A Yoruba translated version of the OLBPDQ was used for participants who were literate in the Yoruba language and preferred the Yoruba version. A Pearson product moment correlation coefficient (r) of 0.86 was obtained for the criterion validity of the back translation of the Yoruba version used in the study.

*Quadruple Visual Analogue Scale* (QVAS) was used to assess pain intensity of the participants [34]. The QVAS assesses pain intensity under four categories, as pain right now, typical or average pain, pain level at its best and pain level at its worst respectively. Pain level is assessed on the scale line marked 0 – 10. Mark 10 stands for most severe pain while mark 0 stands for no pain. The ability of this scale to assess pain under the four different factors gives it an advantage over the other pain tools. For patients with long-term LBP, the average pain grade is often used [34]. A Pearson product moment correlation coefficient (r) of 0.88 was obtained for the criterion validity of the back translation of the Yoruba version used in this study.

The translation of the different questionnaires into the Yoruba language was carried out at the Department of Linguistics and African Languages of Obafemi Awolowo University, Ile Ife, Nigeria.

### Procedures

Each treatment module comprised warm up, main exercise and the cool down phase. The warm-up and the cool-down phase involved a low intensity active stretching of the upper extremities and low back and strolling at self-determined pace around the research venue for about five minutes.

### The McKenzie protocol

The McKenzie extension protocol was exclusively used in this study. The protocol involves a course of specific lumbosacral repeated movements in extension that cause the symptoms to centralize, decrease or abolish [28]. The extension activities include “Extension Lying Prone”, “Extension In Prone”, and “Extension In Standing” [21, 28]. The movement was repeated up to ten times.

### Static back extensors endurance exercise

Static back extensors endurance exercise includes five different exercises of increasing level of difficulty where the positions of the upper and lower limbs were altered [24]. The participants began the exercise training programme with the first exercise position, but progressed to the next exercises at their own pace when they could hold a given position for 10 s. On reaching the fifth progression, they continued with the fifth progression until the end of the exercise programme [24]. The dosage of series of 10 repetitions was used in this study and it was adopted from a previous protocol [25].

### Dynamic back extensors endurance exercise

Dynamic back endurance exercise was a replica of the static back extensors endurance exercise protocol in terms of exercise positions, progressions and duration. However, instead of static posturing of the trunk and limbs in the test positions, the participants moved the trunk and the limbs up to 10 times synchronous to a set metronome count using the Wittner Metronom system Maelzel (Made in Germany).

Irrespective of treatment group, the participants received back care education. The back care education comprised of a 9-item instructional guide on standing, sitting, lifting and other activities of daily living for home exercise (Additional file 1). The details of the treatment procedure for the different protocols have been published elsewhere by the authors [35]. The exercise period ranged between 30 and 45 min for each group. Following the assessment for all participants at inclusion into the study, two additional assessments were made at the 4^th^ and 8^th^ week of the study. This study was conducted over a 12-month period.

### Data analysis

Descriptive statistics of mean and 95 % Confidence Interval (CI) were used to summarize all data. Analysis of Variance (ANOVA) was used to compare demographic variables of the participants in the different treatment groups. Kruskal Wallis test was used to compare the categorical variables such as BBQ, PSEQ and FABQ at baseline in the different treatment groups. Tukey multiple comparisons was used for post-hoc test analysis. Friedman’s ANOVA- (a non-parametric equivalent of the repeated measures ANOVA) was used for within group comparison of the effects of the different treatment regimen on the categorical variables. Wilcoxon signed ranked test was used as the post-hoc multiple comparisons to test for any significant difference found in the Friedman’s F-ratios. Alpha level was set at 0.05. The data analyses were carried out using SPSS 13.0 version software (SPSS Inc., Chicago, Illinois, USA).

## Results

The mean (CI) for age, height, weight and BMI of all the participants was 51.8 (C1:50.0 – 53.6 years), 1.66 (1.65 – 1.67 m), 76.2 (73.5 – 78.9Kg) and 27.2 (26.1 – 28.3 kg/m^2^) respectively. The general and baseline clinical characteristics of the participants by treatment group is presented in Table 1. No significant differences were found among groups with respect to their general and baseline clinical characteristics (*p* > 0.05). Friedman’s ANOVA and Wilcoxon signed ranked test multiple comparisons of psychosocial variables treatment outcomes among participants in the MPG, MPSBEEG, MPDBEEG across the 3 time points of the study is presented in Table 2. Results among the different groups showed that there were significant differences (*p* < 0.05) in the participants’ outcome parameters across the 3 time points of the study. Table 3 shows the Kruskal Wallis test comparison of the participants’ treatment outcomes (mean change) for the psychosocial variables at the 4^th^ and 8^th^ week of the study. There were no significant differences (*p* > 0.05) in mean change of BBQ and FABQ scores across the groups at the end of the 4th week of the study. However, there were significant differences in mean change of PSEQ across the group (*p* < 0.05) at the end of the 4^th^ and 8^th^ week of the study respectively. The Tukey multiple comparisons analysis was used to elucidate where the differences within between groups lie.

**Table 1:** Comparison of the participants’ general characteristics by treatment groups

	MPG	MPSBEEG	MPDBEEG		
Variable	(*n* = 25)	(*n* = 22)	(*n* = 20)		
	Mean (95 %CI)	Mean (95 %CI)	Mean (95 %CI)	F-ratio	p-value*
Age (yr)	50.6 (47.5 – 53.7)	51.2 (47.9 – 54.5)	53.8 (50.6 – 57.0)	1.106	0.339
Height (m)	1.67 (1.65 – 1.69)	1.66 (1.64 – 1.68)	1.68 (1.66 – 1.70)	2.185	0.331
Weight (Kg)	76.3 (72.2 – 80.4)	75.2 (69.3 – 81.1)	77.2 (72.2 – 82.3)	0.156	0.856
BMI (Kg/m^2^)	27.5 (25.8 – 29.2)	27.3 (25.0 – 29.6)	26.9 (25.1 – 28.7)	0.093	0.912
VAS average	6.04 (5.28 – 6.80)	6.54 (5.78 – 7.30)	6.10 (5.26 – 6.94)	1.203	0.307
	Mean rank	Mean rank	Mean rank	H	p-value**
BBQ	28.1	35.1	40.3	4.788	0.091
PSEQ	32.8	33.4	36.3	0.408	0.815
FABQ-P	33.8	35.2	32.9	0.166	0.920
FABQ-W	30.2	35.2	37.5	1.840	0.399
OLBPQ	45.2	41.5	45.3	0.365	0.574

*MPG* McKenzie Protocol Group, *MPSBEEG* McKenzie Protocol plus Static Back Endurance Exercise Group, *MPDBEEG* McKenzie Protocol plus Dynamic Back Endurance Exercise Group, *CI* Confidence Interval, *VAS* Visual Analogue Scale, *BBQ* Back belief questionnaire, *PSEQ* Pain self efficacy questionnaire, *FABQ-P* Fear-Avoidance Beliefs Questionnaire – (Physical), *FABQ-W* Fear-Avoidance Beliefs Questionnaire (Work), *ODI* Oswestry Disability Index

*indicates - One-way ANOVA; **indicates - Kruskal Wallis test

**Table 2:** Psychosocial variables treatment outcomes among participants in the MPG, MPSBEEG, MPDBEEG across the 3 time points of the study

	Baseline	4^th^ week	8^th^ week		
Outcome	Mean rank	Mean rank	Mean rank	χ2	p-value**
MPG (*n* = 25)					
BBQ	28.1^a^	34.4^b^	36.4^b^	49.238	0.001
PSEQ	32.8^a^	44.7^b^	47.1^b^	45.632	0.001
FABQ-P	33.8^a^	14.7^b^	11.1^b^	50.210	0.001
FABQ-W	30.2^a^	24.2^b^	19.3^c^	48.980	0.001
MPSBEEG (*n* = 22)					
BBQ	35.1^a^	36.5^b^	38.1^b^	37.904	0.001
PSEQ	33.4^a^	45.5^b^	47.6^b^	41.302	0.001
FABQ-P	35.2^a^	14.6^b^	11.0^b^	40.000	0.001
FABQ-W	35.2^a^	25.7^b^	21.2^b^	44.000	0.001
MPSBEEG (*n* = 20)					
BBQ	40.3^a^	38.4^b^	39.8^c^	35.096	0.001
PSEQ	36.3^a^	47.6^b^	50.5^c^	38.100	0.001
FABQ-P	32.9^a^	14.8^b^	11.1^c^	39.980	0.001
FABQ-W	37.5^a^	26.8^b^	22.2^c^	40.000	0.001

**indicates - Friedman’s ANOVA

*Superscripts (* ^*a,b,c*^ *)*. Based on the Wilcoxon signed ranked test results, for a particular variable, mean values with different superscript are significantly (*p* < 0.05) different. Mean values with same superscripts are not significantly (*p* > 0.05) different

*BBQ* Back belief questionnaire, *PSEQ* Pain self efficacy questionnaire, *FABQ-P* Fear-Avoidance Beliefs Questionnaire – (Physical), *FABQ-W* Fear-Avoidance Beliefs Questionnaire (Work)

**Table 3:** Comparison of the participants’ treatment outcomes (mean change) for the psychosocial variables at the 4^th^ and 8^th^ week of the study

	MPG	MPSBEEG	MPDBEEG		
	(*n* = 25)	(*n* = 22)	(*n* = 20)		
Outcome	Mean rank	Mean rank	Mean rank	H	p-value**
Week four					
BBQ	29.0	35.8	38.3	3.479	0.176
PSEQ	26.6^a^	36.5^b^	40.5^b^	8.020	0.018
FABQ-P	36.2	31.2	34.3	0.933	0.627
FABQ-W	35.7	27.2	39.4	5.142	0.077
Week eight					
BBQ	34.4	34.9	32.5	0.202	0.904
PSEQ	25.5^a^	37.4^b^	43.5^c^	18.106	0.001
FABQ-P	35.4	31.9	34.6	0.484	0.785
FABQ-W	36.1	28.8	39.6	3.746	0.154

**indicates - Kruskal Wallis test

*Superscripts (* ^*a,b,c*^ *)*. Based on the Tukey multiple comparisons test results, for a particular variable, mean values with different superscript are significantly (*p* < 0.05) different. Mean values with same superscripts are not significantly (*p* > 0.05) different

*BBQ* Back belief questionnaire, *PSEQ* Pain self efficacy questionnaire, *FABQ-P* Fear-Avoidance Beliefs Questionnaire – (Physical), *FABQ-W* Fear-Avoidance Beliefs Questionnaire (Work)

## Discussion

This study investigated the effect of static or dynamic back extensors endurance exercise in combination with MP on psychosocial variables in patients with long-term mechanical LBP. The within-group results from this study revealed that MP alone or in combination with static or dynamic back extensors endurance had significant effects on fear-avoidance behaviour, pain self-efficacy belief and back pain consequences belief respectively. It has been reported that the use of McKenzie’s directional preference approaches significantly and rapidly improved psychosocial outcomes such as depression and work interference in patients with LBP [36]. With the evolution of the yellow flags concepts, psychosocial factors have been acknowledged as important prognostic factors and treatment effect-modifiers-or-mediators in patients with LBP [37–39] but are either inadequately addressed or ignored within standard practice [13]. With the advent and increasing understanding of biopsychosocial model of health, the complex interdependent relationships between the physical and biomedical features of LBP and the psychosocial factors are becoming clear [40]. Consequently, treatment based on the biopsychosocial model that will address biological, social and psychoscial variables in patients with LBP has been advocated [41].

Screening for psychosocial risk factors and subsequently targeting interventions on specific factors are believed to improve patients’ outcome [42–44]. As a result, cognitive behavioural and educational interventions targeting risk factors in patients with long-term pain and disability have been implemented in some studies with varied outcomes ranging from no significant to moderate effect [42–45]. However, given that there is some evidence of benefit for traditionally physical therapy for patients with LBP, the added advantage of integrating psychosocial interventions into the armamentarium of therapeutic options for this group of patients is questionable [40]. Smeets & colleagues [46] submit that active physical therapy regimen primarily designed to improve physiological aspects of LBP such as aerobic fitness level, low back muscle strength and endurance can also reduce the impact of psychosocial factors such as pain catastrophizing that it did not deliberately target. Some other investigators concur that exercise generally has a potential benefit on psychosocial aspect of patient with long-term LBP [47–49]. Hill & Fritz [38] explain that it may not necessarily follow that a psychologist is better placed to improve treatment outcomes than a physical therapist, even when the goal of treatment is the mediation of a psychosocial factor such as pain catastrophizing.

From the between-group result in this study, the effect of MP alone was comparable with the other treatment regimens except for pain self-efficacy belief where the addition of dynamic endurance exercise to MP led to higher treatment effect at the 4^th^ and 8^th^ week respectively. It is adduced that the significant higher treatment outcome in the MPDBEEG might be due to the combined effects of movements and overload stimulus on the back extensor muscles. Specifically, the treatment module for patients in the MPDBEEG contains movement ingredients on double ground. Firstly, from the MP, which is the baseline treatment involving a series of active repeated movements, and secondly, the dynamic back extensors endurance exercise involving repeated movements of the trunk and limbs in the sagittal plane. This finding corroborates previous reports that dynamic endurance training may be needed more than static endurance in patients with LBP, as most of the daily tasks involve dynamic movement [50, 51].

The rationale for the significant improvement in psychosocial outcomes in the different regimens can be associated with movement and activities enhancement component of the interventions. Long-term LBP often results in deconditioning leading to loss of joint motion and inadvertently fear-avoidance behaviour and pain catastrophizing. For patients with LBP, fear-avoidance beliefs signify the potential to inhibit physical activities or movements resulting from cognitive and emotional concerns and fears of provoking pain and further damage to the spine [52]. Elevated fear-avoidance beliefs are most common in long-term LBP and are strongly associated with disability [53, 54]. Pain self-efficacy and fear of movement have been proposed to explain the relationship between pain and disability in patients with long-term LBP [55]. Furthermore, based on the theory of social learning, self-efficacy describes the confidence the person has in his or her own ability to achieve a desired outcome [56]. Therefore, there is an inverse association between levels of self-efficacy and levels of pain and disability in patients with long-term pain [57–59]. The mutual interrelationship between these different psychosocial constructs and pain and disability is complex and intricate. However, these psychosical impairments are likely to change following the resumption of movement and activities despite the presence of pain [60]. Movement is reported to enhance healing in the musculoskeletal system by stretching muscles, tendons and ligaments, by increasing blood and nutrients supply to back extensor muscles, by mobilizing stiff joints, and by mechanically affecting disc pathology, or a combination of all the different effects [23, 61]. The movement component of the treatment regimens as used in this study may have resulted in reconditioning of the patients by making them to expand the limits to their physical functioning, enhance their pain control ability and improve the psychosocial factors affected by LBP. Previous reports suggests that patients with LBP whose treatment regimen do not avoid pain and movements have less disability [62, 63]. Harding and Watson [22] submitted that improvement in overall physical function is linked with improvement in psychosocial function.

From the post-hoc results in this study, it was observed that the different treatment regimens had significant effect at the 4^th^ week on all the psychosocial variables. The effect of the different treatment regimens on the psychosocial variables were significantly higher at the 8^th^ week compared with 4^th^ week of the study. However, the effect of MP alone on beliefs about the consequences of back pain and fear-avoidance behaviour (physical) were comparable at the 4^th^ and 8^th^ week of the study. Furthermore, the effect of MP and static back endurance exercise on beliefs about the consequences of back pain, pain-self efficacy belief, and fear-avoidance behaviour (physical and work) were comparable at the 4^th^ and 8^th^ week of the study. It was adduced that a four-week MP as well as the addition of static or dynamic back extensors endurance exercises are effective in improving psychosocial variables of patients with long-term mechanical LBP. Combining static and dynamic back extensors endurance exercise with the MP is recommended in improving psychosocial factors in patients with long-term mechanical LBP. When considering the number of sessions (3 sessions per week for 8 weeks –totaling 24 sessions), the treatment regimens in this study is considered quite extensive and a high dose [64, 65].

This current study has some potential limitations. Firstly, the study was carried out among patients with directional preference for extension only and the finding cannot be generalized to other patients’ population with directional preference for flexion or lateral or none at all based on the McKenzie assessment. There was also lack of a ‘no exercise’ control group (for ethical reasons) and no longer term follow-up. In addition, we did not conduct an intention-to-treat analysis, despite the drop-outs percentage. However, a majority of the patients that dropped-out, absconded due to improvement in their health condition than any other reason. Nonetheless, it is assumed that the treatment outcomes obtained from this study are due to the effect of the interventions. The assertion is based on the no significant differences observed in physical characteristics and baseline psychosocial parameters of the patients in the different treatment groups. Baseline characteristics of participants in clinical trials for LBP are often considered to be significant mediators of treatment effects when there is a significant difference between groups. Comparable baseline characteristics in clinical trials are reported to decrease the chances of co-founders on treatment effects.

## Conclusions

The McKenzie protocol alone or its combination with either static or dynamic back extensor muscles endurance exercise have comparable effect on beliefs about the consequences of back pain and fear-avoidance belief behaviour in patients with long-term mechanical LBP. The addition of dynamic endurance exercise to the McKenzie protocol led to significantly higher positive effects on the psychosocial variables of pain self-efficacy belief in patients with long-term mechanical LBP.

## Additional file

Additional file 1:
**Illustration pamphlet for your back care.** (DOCX 374 kb)

## Abbreviations

BBQ: Back Belief Questionnaire
BPCB: Back Pain Consequences Belief
CONSORT: Consolidated Standards of Reporting Trials
FAB: Fear-Avoidance Behaviour
FABQ-P: Fear-Avoidance Beliefs Questionnaire – (Physical)
FABQ-W: Fear-Avoidance Beliefs Questionnaire (Work)
LBP: Low-Back Pain
MILSAF: McKenzie Institute’s Lumbar Spine Assessment Format
MPDBEEG: McKenzie Protocol plus Dynamic Back Endurance Exercise Group
MPG: McKenzie Protocol Group
MPSBEEG: McKenzie Protocol plus Static Back Endurance Exercise Group
OLBPQ: Oswestry Low Back Pain Questionnaire
PSEB: Pain Self-Efficacy Belief
PSEQ: Pain Self Efficacy Questionnaire
