# Supplementary material for: Comparative efficacy of three active treatment modules on psychosocial variables in patients with long-term mechanical low-back pain: a randomized-controlled trial
Source: Arch Physiother. 2015 Sep 21;5:10. doi: 10.1186/s40945-015-0010-0 (PMC5759898; doi:10.1186/s40945-015-0010-0)
Supplement: Additional file 1: — Illustration pamphlet for your back care. (DOCX 374 kb) [file 40945_2015_10_MOESM1_ESM.docx › ESM/40945_2015_10_MOESM1_ESM.docx]

**ILLUSTRATION PAMPHLET FOR YOUR BACK CARE**

**Please follow the instructions below carefully:**

1. Avoid prolong sitting


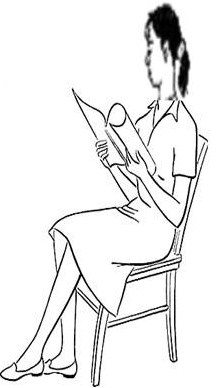


1. Avoid bending

**
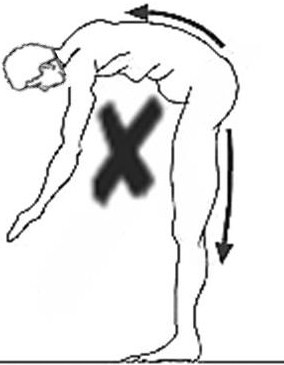
**

1. Avoid stooping


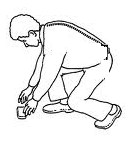


1. Avoid squatting


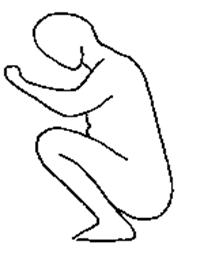


1. Interrupt static posture every thirty minutes before developing any discomfort


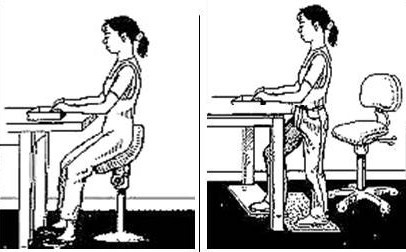


6. Maintain lumbar lordosis (hollow in the low back) in sitting and other postures.


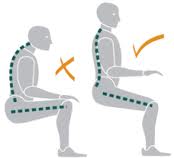

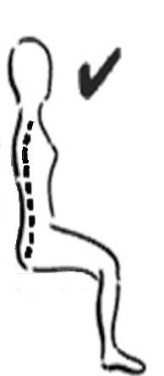


7. Use supportive roll/cushion placed in the hollow of the back in sitting position


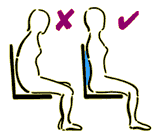


1. Avoid sitting on low chairs, stool and soft couch with deep seat as much as possible.


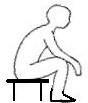


1. Use a firm, high chair with a good comfortable back support;


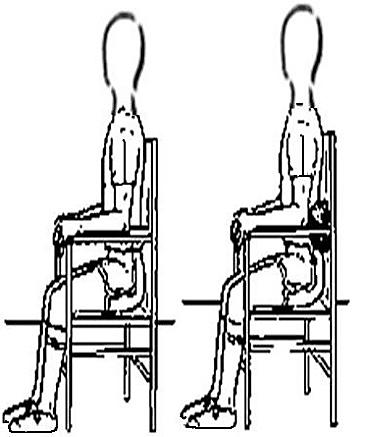


1. Consciously control and maintain good upright posture when sitting on a seat without back rest or support


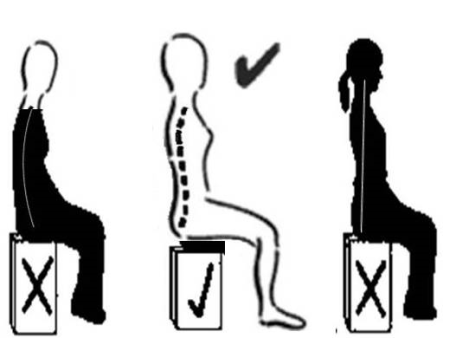


1. Avoid lifting a heavy load as much as possible - when you have to lift, carry only a moderate load.


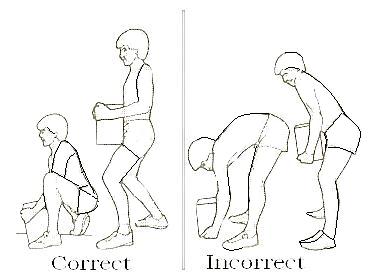


1. Carry out your back exercises daily - bend backward five (5) times with hand placed in the hollow of your back every two hour


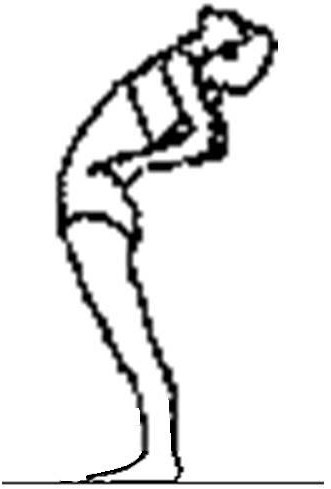


**N.B**: **Regularly look through the postal illustrations to remind yourself of what you need to do**.

**FÚN IGBÀÁDÙN ÈYÌN ÀTI ÀLÁÁFÍÀ RE, TỆLE ÀWON ÌLÀNÀ TÓ WÀ NÍSÀLỆ YÍÌ**

1. Yera fún ìjókòó pípé,


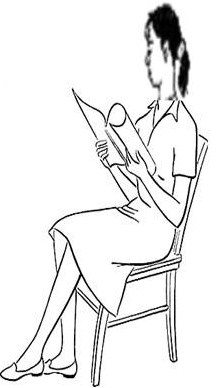


1. Yera fún bíbèrè mólè

**
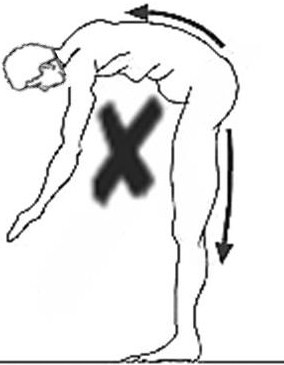
**

1. Yera fún ìlósòó


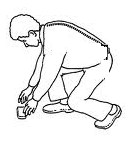


1. Yera fún títiro


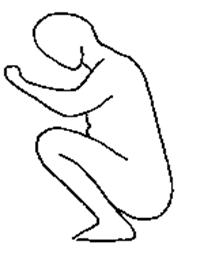


1. Máa pàrò ipò ara léhìn ogbòn ìséjú tí o bá jókòó, kú ara tó ni ó.


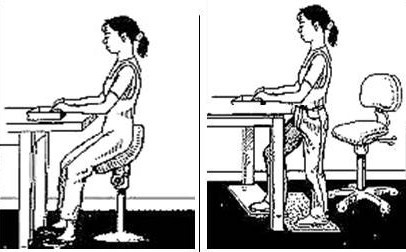


6. Jé kí eegun ìbàdìí re nàró tí o bá jókòó.


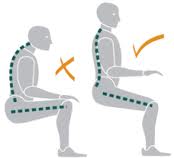

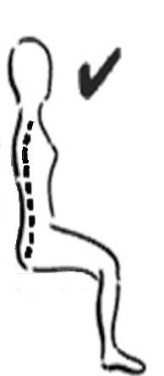


7. Fi nnkan to fuye ti ehin re ti o ba jókòó


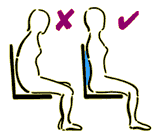


8. Yera fún jijókò lóri àga tí kò ga, àpóti àti ijókò tó rọ ti ò niho


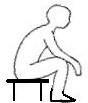


9. Lo àga tó lágbára, tí ó ga, tí ó sí nì ìfèhìntì.


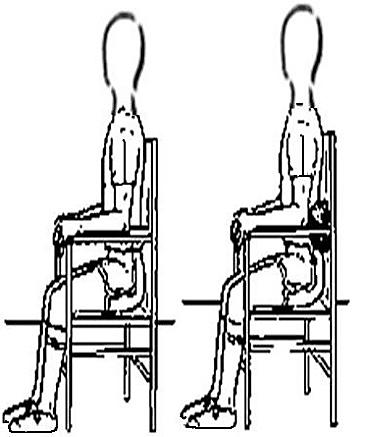


10. Mòómò jókòó dáadáa nígbàkugbà tí o kò bá lo ohun ìtì èhìn.


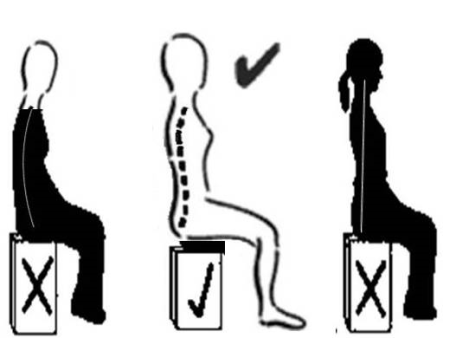


11. Sóra fún gbígbé erù wúwo-nígbà tí ó bá pon dandan, gbé ìwònba.


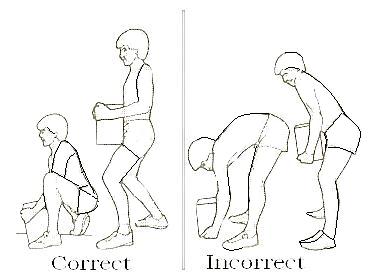


1. Máa se eré amárale fún èyìn l’ójojúmó, tè s’èhìn léémáàrún-wò, nípa fífi ọwó te ìbàráàdí s’íwájú ní wákàátí méjì-méjì


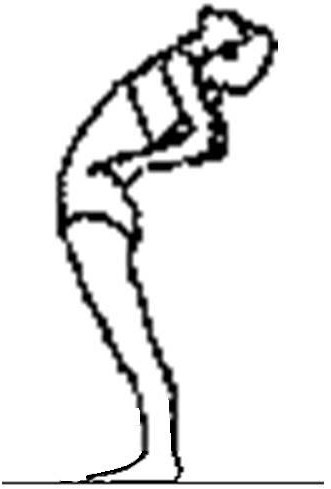


**Àkíyèsí p**à**t**à**kì**: Máa wo àwòrán asàpèjúwe ní gbogbo ìgbà láti rán ara rèe létí àwon ohun tí o ní láti se.
